# Jet Ventilation during Rigid Bronchoscopy in Adults: A Focused Review

**DOI:** 10.1155/2016/4234861

**Published:** 2016-10-26

**Authors:** Laurie Putz, Alain Mayné, Anne-Sophie Dincq

**Affiliations:** Department of Anesthesiology, Université catholique de Louvain, CHU UCL Namur, Avenue G. Therasse 1, 5530 Yvoir, Belgium

## Abstract

The indications for rigid bronchoscopy for interventional pulmonology have increased and include stent placements and transbronchial cryobiopsy procedures. The shared airway between anesthesiologist and pulmonologist and the open airway system, requiring specific ventilation techniques such as jet ventilation, need a good understanding of the procedure to reduce potentially harmful complications. Appropriate adjustment of the ventilator settings including pause pressure and peak inspiratory pressure reduces the risk of barotrauma. High frequency jet ventilation allows adequate oxygenation and carbon dioxide removal even in cases of tracheal stenosis up to frequencies of around 150 min^−1^; however, in an in vivo animal model, high frequency jet ventilation along with normal frequency jet ventilation (superimposed high frequency jet ventilation) has been shown to improve oxygenation by increasing lung volume and carbon dioxide removal by increasing tidal volume across a large spectrum of frequencies without increasing barotrauma. General anesthesia with a continuous, intravenous, short-acting agent is safe and effective during rigid bronchoscopy procedures.

## 1. Introduction

Rigid bronchoscopy (RB) allows diagnostic and therapeutic procedures to be performed in the tracheobronchial tree. This technique includes specific features such a common work field for the anesthesiologist and the interventional pulmonologist and also special management of the airway, which is often already compromised. The introduction of the Venturi jet injector technique by Sanders in 1967 allowed the achievement of the goal of adequate ventilation and an open access to the airway. In this focused review, we discuss jet ventilation used during procedures realized with a rigid bronchoscope including superimposed high frequency jet ventilation.

## 2. Jet Ventilation

The term jet describes a rapid flow of compressed gas delivered via a nozzle. This stream is delivered at high pressure (0.3–3 bar) (all pressure measurements are expressed in bar or mbar as on the front screen of the jet ventilator equipment (converting from measurements in pascal (Pa) as required)). High frequency jet ventilation (HFJV) is defined as ventilation involving a high pressure jet at a supraphysiological frequency between 120 and 600 impulses min^−1^ followed by a passive expiration. At present, manual techniques are tending to be replaced by mechanical jet respiration. The HFJV ventilator is electrically powered and is supplied with compressed air and oxygen from the hospital supply system. After passing a pressure regulator, the gas mixture reaches a manifold of four electromagnetic solenoid valves. These valves are closed in the resting position. Once a valve is opened, a small gas volume is released. This ventilation technique allows the delivery of a small single dose of gas with a tidal volume between 1 and 3 ml/kg of body weight. Low frequency jet ventilation (LFJV) or normofrequent jet ventilation provides ventilation at frequencies between 10 and 30/min^−1^. A combination of HFJV and LFJV is called superimposed high frequency jet ventilation (SHFJV) and is a double-jet technique. Several jet ventilators are available such as Monsoon III, Acutronic®, Switzerland, or TwinStream®, Carl Reiner GmbH, Austria.

In view of the extremely small tidal volume experienced during jet ventilation, which is smaller than the total dead space (anatomical + equipment), it can be difficult to predict the response in terms of classic human respiratory physiology. For example, gas exchange may result not only from standard bulk flow (convection) ventilation occurring during a normal respiratory cycle where the gas is directly injected into the alveoli but also from a combination of four gas stream mechanisms, namely, laminar flow, pendelluft effect, Taylor dispersion, and cardiogenic mixing (not covered in this review). Alveolar ventilation (the portion of gas taking part in gas exchange) depends on the volume delivered by the injector and the entrained volume (called the Venturi effect) minus the reflux volume (which is often insignificant in clinical practice except in the case of poor airway compliance leading to a smaller minute volume delivered to the alveoli). This entrained air is greater when the driving pressure is high. The very short injector catheter being placed within the side arm of the base of the rigid bronchoscope, in a very proximal position, leads to a major dilution of the injected gas by entrainment of ambient air. Consequently, if 100% oxygen is used as the jet gas, the effective FiO_2_ in the trachea will be 0.8–0.9 [1]. Pneumonia and active asthma are absolute contraindications to both techniques because overdistension and/or hypoventilation occur in the presence of increased respiratory compliance or elevated bronchial resistance [2]. Inequality of pulmonary compliance provokes alveolar hyperinflation in certain localizations and nonventilation at others. Other contraindications to jet ventilation are all relative and include morbid obesity if patients have poor basal oxygenation [3], uncontrolled coagulopathy, and tracheal obstruction for the less experienced operator.

## 3. Rigid Bronchoscopy

Rigid bronchoscopy (RB), a technique for interventional pulmonology performed under general anesthesia with an increasingly wide range of indications from debulking tumors to placing tracheobronchial stents, can benefit from jet ventilation techniques. These ventilation techniques enhance technical access and adequate ventilation during procedures [4]. A rigid bronchoscope consists of a metal tube fitted onto a base which has three access points (or entrances). The first is the* wide axial access* dedicated to the optical device including flexible or rigid fiberscope and instruments such as clips, dilatation balloons, or stents, as required. An integrated video system allows visualization of the work field by each practitioner. The second is an* oblique lateral entrance* dedicated to specific devices such as a laser fiber, an argon plasma coagulation probe, or a suction cannula. The third is a* lateral entrance* specifically devoted to ventilation.

## 4. Settings

A skill must be acquired by theoretical instruction, medical simulator, and a more frequently use in routine practice. Then a careful adjustment of the settings and alarms of airway pressure are essential because incorrect use can lead to very harmful consequences such as barotrauma or cardiovascular collapse [3]. Three parameters influence the delivered gas volume: frequency (*F*) expressed in counts per minute (CPM) between 60 and 600 min^−1^, inspiration time (*IT*): the percentage (%) of total cycle time spent in inspiration, and driving pressure (*DP*) around 0.3–3 bar. DP is also called injection pressure. Two alarms must be adjusted before using HFJV: pause pressure (*PP*) and high peak inspiratory pressure (*PIP*). It should be kept in mind that this is an open ventilation system and therefore does not require an expiration valve. This open system is a serious advantage for the lung specialist because he can change its instruments as he wants without stress. This is possible due to the fact that the system has to continuously remain open to the atmosphere. A consequence of this is that the expiratory pathway (in this case, the rigid bronchoscope) cannot be obstructed. PP is measured from the tip of the HFJV catheter, reflecting the pressure inside the airway, approximating the mean airway pressure. When the preset value is reached, the alarm is activated and further inspiration is suspended until the set limit is reached (this is also called end-expiratory pressure: EEP set limit or high limit for positive end-expiratory pressure (PEEP)). Jiang and Kacmarek suggest a value of less than 10 cm H_2_O, particularly in the case of moderate-to-severe tracheal stenosis where high auto-PEEP can rapidly increase due to compromised expiration [5]. While PIP is reached, the next inspiration is not engaged. Informative data are provided in Table 1. It seems to be important to begin at the lower value and after that to increase parameters depending on PetCO_2_ or PaCO_2_, chest expansion, saturation, and clinical observations (color, auscultation) requiring a permanent presence of the anesthesiologist. Eventually, arterial gas sampling is used to guide ventilation in highly symptomatic patients. The preset* bypass* allows a fresh gas flow between 0 and 70 l/min. It is useful for manual ventilation at the time of induction or improving ventilation during the procedure by delivering manual positive pressure ventilation. To be most effective the operator should use two hands to form an airtight seal around the mouth while someone else delivers positive manual pressure ventilation via the side arm of the rigid bronchoscope. The inspired fraction of oxygen (FiO_2_) is also adjustable. A value below 0.4 is recommended during laser procedures to prevent combustion [3]. Laser procedures should only be started when this value is reached and confirmed on the front panel of the jet ventilator. Capnography is provided by some jet ventilators: the jet ventilator gives 5 low frequency inspirations and then the analysis can be performed through the RB. Adjustable levels of humidification of the fresh gas flow (between 0 and 100 percent) allow protection against dryness of the airway mucosa and damage to the ciliary function [6]. A high level of humidification can lead to an increase in airway resistance. We recommend a progressive increase of the humidification level, achieved using sterile water. Saline is prohibited due to the fact that sodium and chlorine ions can clog the ventilator. In certain ventilators, fresh gas flow can also be rewarmed. This feature can be particularly important in children and severely compromised patients. In cases of bronchopleural fistula, the clinical state is characterized by leakage around the fistula. The following setting have been suggested (from experiments using an animal model) in order to reduce leakage in such settings: ventilator rate > 200 min^−1^, DP at 1.5 bar or less, and an injection time around 40% [7].

## 5. Superimposed Jet Ventilation

**Figure 1:**
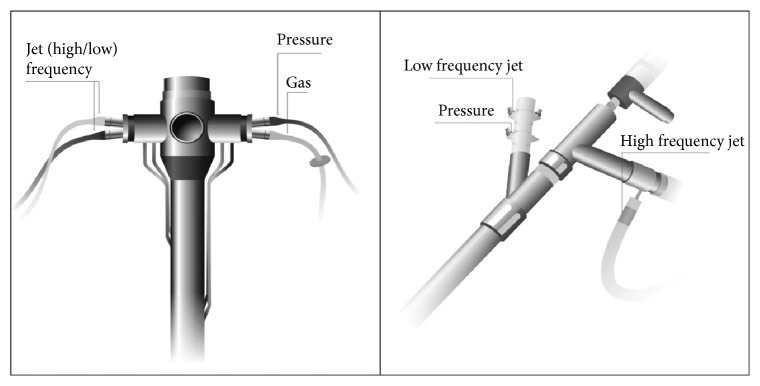
Type of devices allowing SHFJV.

This ventilation technique (SHFJV), which uses a combination of HFJV and LFJV [8], offers numerous benefits and is interesting to explore. SHFJV has been shown to be safe and effective in clinical practice, for example, in endoscopic laryngotracheal surgery in patients with severe tracheal stenosis. This technique creates optimal conditions for such surgery, including laser treatment and stent implantation [9]. Even in the presence of serious airway obstruction, SHFJV has been shown to maintain adequate PO_2_ and carbon dioxide (CO_2_) removal [10, 11]. The goal of including LFJV is to increase minute ventilation and improved CO_2_ removal [12] by improving lung recruitment leading to better oxygenation [13]. Sütterlin et al., using a porcine model without respiratory disease, compared SHFJV and single-frequency HFJV in the absence of airway obstruction and concluded that both techniques offer adequate ventilation, except for single-frequency HFJV frequencies ≥ 300 min^−1^ [14]. Over this range, tidal volume approaches the expected dead space resulting in poor gas exchange and convection representing the bulk of gas exchange in this model (where jet ventilation is provided directly via a cuffed endotracheal tube and gas exchange results from a combination of convection and diffusion) is reduced [5, 15]. Furthermore, SHFJV was more effective in increasing end-expiratory volume than single-frequency HFJV [14]. Secondly, once a tracheal stenosis was added to this model, single-frequency HFJV at a frequency not exceeding 150 min^−1^ provided adequate CO_2_ removal and oxygenation even in cases of severe airway obstruction (75% obstruction for the 4 mm stent) while for SHFJV, the frequency of the HFJV component of the SHFJV had little impact. In cases of severe stenosis, SHFJV improved gas exchange and lung volume which can be beneficial [16]. In clinical practice, tracheal stenosis is not the same as a fixed stent, so airflow due to SHFJV may produce shear and stress resulting in edema increasing the severity of the stenosis [5] and clinicians must be alert to this potential harmful event. The various marketed systems for SHFJV are either incorporated permanently in the rigid bronchoscope or attached to a standard bronchoscope (e.g., Efer-Dumon) (Figure 1). It is interesting to know that, during SHFJV, PP is inhibited by some ventilators (e.g., Monsoon III, Acutronic®, Switzerland) making the PIP adjustment essential. Hence, at the beginning of the procedure and during all of this, it is really essential to adapt the values of this PIP. As regards the settings, DP and/or* I* :* E* ratio of each jet component, little is known due to the complex interplay between the 2 components. Further studies are needed to gain understanding in this field [17].

## 6. Complications

While jet ventilation offers numerous advantages, a number of potential complications must be kept in mind. These may be ameliorated with a good knowledge a cardiorespiratory physiology, patient specific pathology, and appropriate use of the jet ventilator equipment in terms of operation and alarm settings. Major complications include barotrauma such as pneumothorax, pneumomediastinum, subcutaneous emphysema, and inadequate gas exchange (hypoxemia, hypercapnia) mostly in patients with severe lung disease particularly restrictive pathology [1] or circulatory collapse. The presence of airway narrowing is a risk factor [18] and must be only managed by experienced anesthesiologists. Cheng et al. found that acute moderate CO_2_ retention (60–100 mmHg) was not associated with serious consequences among patients receiving RB; conversely severe hypercapnia (>100 mmHg) was associated with lower levels of PaO_2_ [19]. Poor physical status (American Society of Anesthesiologists (ASA) grade IV) and baseline oxygen saturation of 95% or less are predictive factors of complications such as hypercapnia, hypoxemia, and hemodynamic instability during RB with HFJV [20]. To strive to normocapnia, settings adjustments of HFJV are provided in Table 2. In case of hypoxemia, plus a careful auscultation, settings of HFJV must be adapted following Table 2. However, we must not forget that the first cause of hypoxemia is the lateralization of the RB leading to single-lung ventilation, a proper adjustment of the RB into the trachea can greatly reduce this problem. Other causes include pneumothorax or bronchial hyperreactivity; specific treatments such as thoracic drainage or aerosols must be applied. Nevertheless, in case of persistent hypoxemia, manual ventilation must be envisaged and even orotracheal intubation. In case of life threatening situation, extracorporeal membrane oxygenator can be used as rescue treatment [21]. Malposition of a rigid bronchoscope into the digestive tract leading to gastric rupture or distension can easily be avoided by performing a flexible fiberoptic bronchoscopy to confirm the correct position in the airway before starting jet ventilation. Anesthesiologists should be aware of direct complications related to RB procedures including tooth damage during rigid bronchoscope insertion, gas embolism, bleeding, bronchial rupture [22]. If a major bleeding occurs during the procedure, a Fogarty catheter or a bronchial blocker can be inserted to protect the contralateral lung; this technique can also be used preventively during cryobiopsies [23].

## 7. Anesthesia Technique

For increasingly complex procedures [18], RB under general anesthesia (GA) is safe and effective [24] and provides excellent working conditions for the interventional pulmonologist. This may reduce perioperative stress and coughing, provide amnesia without compromising airway patency, and ensure adequate oxygenation during the procedure [25]. GA also provides better hemodynamic stability and patient comfort and shortens the duration of the procedure compared with moderate sedation [26]. Currently, GA with adequate muscle relaxation before rigid bronchoscope insertion and short-acting anesthetic agents has become the common standard for jet ventilation [27]. Since RB is an open airway system, total intravenous anesthesia with short-acting agents (e.g., remifentanil, propofol, and rocuronium) is recommended [22]. The best approach to administer propofol and probably remifentanil is via Target Control Infusion (TCI) mode. Standard monitoring comprises electrocardiography, pulse oximetry, noninvasive blood pressure monitoring, and neuromuscular transmission monitoring. While deep muscle relaxation (posttetanic count 1) ensures excellent conditions during the entire procedure, it must be completely reversed before recovery. Sugammadex at a dose of 4 mg/kg assures a fast, predictable, and safe reversal of the neuromuscular block at the time of rigid bronchoscope removal [28]. Invasive blood pressure monitoring may be required depending on patient comorbidities and/or the bleeding risk from the procedure. Monitoring of depth of anesthesia is useful (bispectral index between 40 and 60), to the extent that RB is considered to create a risk of intraprocedural awareness [29, 30]. This monitoring suffered from misinterpretations due to manipulation of rigid bronchoscope, mechanical artifacts, and/or electronic noise and its result should not be interpreted alone to judge the level of hypnosis but in conjunction with other available clinical signs. The expiration fraction of CO_2_ is often compromised due to the ventilation method, even though new generation jet ventilators include a CO_2_ analyzer. This measurement is carried out on request: the ventilator performs 5 higher tidal volumes at low frequency, followed by an apnea during which the sample is taken. There is a significant gradient between PetCO_2_ and PaCO_2_ due to the fact that the extraction catheter is proximal on the RB. Other alternatives are intermittent blood sample drawing and analysis via an arterial catheter which however remain an invasive technique burdened with complications [31] or transcutaneous measurements. Transcutaneous pressure of CO_2_ (PtcCO_2_) allows a reliable, noninvasive, continuous, simple, and rapid estimation of PaCO_2_ [32]. PtcCO_2_ measurement executed via an electrode is based on CO_2_ diffusion through human tissues. A local hyperemia, obtained by heating skin at 42°C, allows an arterialisation of cutaneous capillaries. It must be emphasized that currently, after a stabilization time between 5 and 10 minutes, it takes only 5 to 10 seconds to obtain a stable signal (reaction time) due to developments in signal digitalization [33]. This technique shows also some limits such as technical factors (e.g., improper application of the sensor, trapped air bubbles in the electrolyte solution, damage to the membrane, and improper calibration) and/or patient factors (e.g., hypoperfusion due to vasoconstriction, hypothermia, shock, low cardiac output or local venostasis, edema, or increased skin thickness) [34].

Decompression of medical gas results in gas temperatures below 15 degrees which can induce hypothermia. To prevent this phenomenon, new ventilators offer warming and humidification of the delivered gas. It is also important to emphasize the indispensable interactive collaboration between healthcare practitioners involved in such procedures. The dedicated personnel must be familiar with the operating environment, the equipment, and the technique to assure patient safety during this potentially dangerous procedure [35].

## 8. Conclusion

Jet ventilation allows the performance of numerous procedures under rigid bronchoscopy. A good knowledge of ventilator use is a vital requirement to reduce avoidable and potentially severe consequences such as barotrauma. A meticulous adjustment of the alarm setting including PP and PIP must be done before each case. SHFJV, teaming up HFJV and LFJV, during laryngotracheal surgery helps to maintain adequate PO_2_ and carbon dioxide removal. The SHFJV by increasing minute ventilation has been shown to improve CO_2_ removal in cases of severe tracheal stenosis in a healthy animal model across a wide spectrum of frequencies of HFJV and also to provide adequate oxygenation by increasing lung volume. At frequencies > 150 min^−1^, single-frequency HFJV efficacy is insufficient to maintain adequate gas exchange. Most procedures are performed under general anesthesia using short-acting intravenous anesthetic agents and muscle relaxants.

## Figures and Tables

**Table 1:** Recommended settings for typical situations.

		SHFJV	
	HFJV	HFJV	LFJV
DP (bar)	0.3–3	0.3–3	0.8–1.5
F (CPM)	120–180	120–600	8–12
IT (%)	0.3–0.4	0.3–0.4	0.3–0.4
PP (mbar)	5–10	5–10	5–10
PIP (mbar)	20	20	20

**Table 2:** Settings adjustments of HFJV component in case of hypoxemia and/or hypercapnia.

	Hypoxemia	Hypercapnia
DP (bar)	↑	↑
F (CPM)	—	↓
IT (%)	↑	↑
FiO_2_	↑	(↑ si PaCO_2_ > 100 mmHg)

## References

[1] Evans E., Biro P., Bedforth N. (2007). Jet ventilation. *Continuing Education in Anaesthesia*.

[2] Rouby J.-J., Viars P. (1989). Clinical use of high frequency ventilation. *Acta Anaesthesiologica Scandinavica, Supplement*.

[3] Bourgain J.-L., Chollet M., Fischler M., Gueret G., Mayne A. (2010). Guide for the use of jet-ventilation during ENT and oral surgery. *Annales Francaises d'Anesthesie et de Reanimation*.

[4] Pawlowski J. (2013). Anesthetic considerations for interventional pulmonary procedures. *Current Opinion in Anaesthesiology*.

[5] Jiang Y., Kacmarek R. M. (2015). Efficacy of superimposed high-frequency jet ventilation applied to variable degrees of tracheal Stenosis: one step forward to optimized patient care. *Anesthesiology*.

[6] Kraincuk P., Kepka A., Ihra G., Schabernig C., Aloy A. (1999). A new prototype of an electronic jet-ventilator and its humidification system. *Critical Care*.

[7] Wood M. J., Lin E. S., Thompson J. P. (2014). Flow dynamics using high-frequency jet ventilation in a model of bronchopleural fistula. *British Journal of Anaesthesia*.

[8] Ihra G., Hieber C., Schabernig C. (1999). Supralaryngeal tubeless combined high-frequency jet ventilation for laser surgery of the larynx and trachea. *British Journal of Anaesthesia*.

[9] Rezaie-Majd A., Bigenzahn W., Denk D.-M. (2006). Superimposed high-frequency jet ventilation (SHFJV) for endoscopic laryngotracheal surgery in more than 1500 patients. *British Journal of Anaesthesia*.

[10] Schragl E., Donner A., Grasl M. C., Kashanipour A., Aloy A. (1995). Ventilation during tracheotomy in extensive, 90% laryngeal stenosis using superimposed high frequency jet ventilation via the jet laryngoscope. *Laryngorhinootologie*.

[11] Schragl E., Donner A., Kashanipour A., Gradwohl I., Ullrich R., Aloy A. (1994). Anesthesia in acute respiratory tract obstructions caused by high degree laryngeal and tracheobronchial stenoses. *Anasthesiologie, Intensivmedizin, Notfallmedizin, Schmerztherapie*.

[12] Bacher A., Pichler K., Aloy A. (2000). Supraglottic combined frequency jet ventilation versus subglottic monofrequent jet ventilation in patients undergoing microlaryngeal surgery. *Anesthesia & Analgesia*.

[13] Kraincuk P., Körmöczi G., Prokop M., Ihra G., Aloy A. (2003). Alveolar recruitment of atelectasis under combined high-frequency jet ventilation: a computed tomography study. *Intensive Care Medicine*.

[14] Sütterlin R., Priori R., Larsson A., Lomauro A., Frykholm P., Aliverti A. (2014). Frequency dependence of lung volume changes during superimposed high-frequency jet ventilation and high-frequency jet ventilation. *British Journal of Anaesthesia*.

[15] Lin E. S., Jones M. J., Mottram S. D., Smith B. E., Smith G. (1990). Relationship between resonance and gas exchange during high frequency jet ventilation. *British Journal of Anaesthesia*.

[16] Sütterlin R., LoMauro A., Gandolfi S. (2015). Influence of tracheal obstruction on the efficacy of superimposed high-frequency jet ventilation and single-frequency jet ventilation. *Anesthesiology*.

[17] Sütterlin R. https://uu.diva-portal.org/smash/get/diva2:735634/FULLTEXT01.pdf.

[18] Goudra B. G., Singh P. M., Borle A., Farid N., Harris K. (2015). Anesthesia for advanced bronchoscopic procedures: state-of-the-art review. *Lung*.

[19] Cheng Q., Zhang J., Wang H., Zhang R., Yue Y., Li L. (2015). Effect of acute hypercapnia on outcomes and predictive risk factors for complications among patients receiving bronchoscopic interventions under general anesthesia. *PLoS ONE*.

[20] Fernandez-Bustamante A., Ibañez V., Alfaro J. J. (2006). High-frequency jet ventilation in interventional bronchoscopy: factors with predictive value on high-frequency jet ventilation complications. *Journal of Clinical Anesthesia*.

[21] Gourdin M., Dransart C., Delaunois L., Louagie Y. A. G., Gruslin A., Dubois P. (2012). Use of venovenous extracorporeal membrane oxygenation under regional anesthesia for a high-risk rigid bronchoscopy. *Journal of Cardiothoracic and Vascular Anesthesia*.

[22] Dincq A.-S., Gourdin M., Collard E. (2014). Anesthesia for adult rigid bronchoscopy. *Acta Anaesthesiologica Belgica*.

[23] Sastre J. A., Cordovilla R., Jiménez M. F., López T. (2014). Management of a transbronchial cryobiopsy using the i-gel® airway and the Arndt endobronchial blocker. *Canadian Journal of Anesthesia*.

[24] Brodsky J. B. (2003). Bronchoscopic procedures for central airway obstruction. *Journal of Cardiothoracic and Vascular Anesthesia*.

[25] Gasparini S. (2011). It is time for patients to undergo bronchoscopy without discomfort. *European Respiratory Journal*.

[26] Jose R. J., Shaefi S., Navani N. (2014). Anesthesia for bronchoscopy. *Current Opinion in Anaesthesiology*.

[27] Sarkiss M. (2011). Anesthesia for bronchoscopy and interventional pulmonology: from moderate sedation to jet ventilation. *Current Opinion in Pulmonary Medicine*.

[28] Dubois P. E., Mulier J. P. (2013). A review of the interest of sugammadex for deep neuromuscular blockade management in Belgium. *Acta Anaesthesiologica Belgica*.

[29] Myles P. S., Leslie K., McNeil J., Forbes A., Chan M. T. V. (2004). Bispectral index monitoring to prevent awareness during anaesthesia: the B-Aware randomised controlled trial. *The Lancet*.

[30] Moerman N., Bonke B., Oosting J. (1993). Awareness and recall during general anesthesia: facts and feelings. *Anesthesiology*.

[31] Okeson G. C., Wulbrecht P. H. (1998). The safety of brachial artery puncture for arterial blood sampling. *Chest*.

[32] Gancel P. -E., Masson R., Du Cheyron D., Roupie E., Lofaso F., Terzi N. (2012). PCO_2_ transcutanée: pourquoi, comment et pour qui ?. *Réanimation*.

[33] Kocher S., Rohling R., Tschupp A. (2004). Performance of a digital PCO_2_/SpO_2_ ear sensor. *Journal of Clinical Monitoring and Computing*.

[34] Huttmann S. E., Windisch W., Storre J. H. (2014). Techniques for the measurement and monitoring of carbon dioxide in the blood. *Annals of the American Thoracic Society*.

[35] Ernst A., Silvestri G. A., Johnstone D. (2003). Interventional pulmonary procedures: guidelines from the American College of Chest Physicians. *Chest*.

